# Technique of Oral Prophylaxis

**Published:** 1915-05

**Authors:** Arthur Day

**Affiliations:** Toronto


### TECHNIQUE OF ORAL PROPHYLAXIS.

ARTHUR DAY, D.D.S., TORONTO.

The technique of oral prophylaxis depends somewhat on the financial position of the patient. The following technique applies to the class of patients who are not rich (stenographers, bookkeepers and those who have fairly good positions), who in deciding if they will have prophylaxis at regular intervals have also to consider whether or not they can afford it. The class, let us say, who can afford four one-hour treatments a year at a fee of from sixteen to twenty dollars per year.

It must be remembered all through the operation that no time can be lost if a prophylaxis which is done only every three months is to be performed efficiently in one hour. The only way to attain efficiency in the above class of patients is to have system in the procedure, and a minimum of waste time. These considerations make an assistant absolutely necessary.

The technique, in the order in which it is performed, is as follows: Blow out all soft matter from between teeth with spray; then pick out any soft matter left with explorer. Remove all tartar, trim off all overhanging edges of fillings and polish fillings having rough and pitted surfaces. Trim cervical edge of gold crowns that do not fit properly, so they do not impinge on the gum. These operations may sometimes require the whole appointment, but they are necessary. In such cases the polishing of the teeth has to be postponed until the next appointment—three months.

While teeth are being thoroughly scaled, the assistant gets ready the outfit of instruments, etc., for the polishing process. This consists of an orange wood stick whittled to a thin spatula-shaped point; two double-end Kuroris holders, one fitted with flat points and one with round points; Cutter's floss silk, sizes R and P; linen strips, a small glass of moistened pumice flour and one of disclosing solution. The assistant should also keep all these renewed as occasion demands during the operation.

Apply disclosing solution to the labial and buccal surfaces of one-half of the upper teeth—the right or left—holding the lip out of the way. Spray solution off the teeth. If the remaining stain shows on the entire surface of all these teeth, go over the teeth with the engine. (This is necessary if the prophylaxis is to be done in one hour.) In ordinary cases polish off stain that remains with flour of pumice carried on a wooden point. For the incisors the orange wood stick; for the posteriors a Kuroris flat point carried in a Kuroris handle. The Kuroris points should have the corners filed off so they will not injure the gum. The point in polishing should be moved in the same direction as the gum line; if the movement is towards and from the gum, powder is apt to be packed under the gum margin. The points used should not have the flattened surface concave shaped, but should be flat, so that the greatest possible area of point is in contact with the tooth while polishing. Nor should they be of too hard a wood. A medium soft wood becomes loaded with the powder better. It is for these reasons that a Kuroris point is recommended in preference to an ivory point. The proximal surface of the tooth is polished with the pointed stick, filed small enough to fit in the approximal space.

The remaining powder is, after the polishing, cleaned off the teeth with the spray. The direction of the spray should be such as not to blow the powder under the gum margin. If only three or four teeth are to be cleaned of powder, apply disclosing solution to the next teeth to be worked on before spraying off powder, and then the one spraying and spitting-out does for both. After going over labial and lingual surfaces of uppers, polish approximal surfaces with Cutter's floss silk. Pass as wide a piece of floss as will fit into each approximal space. After it is placed have assistant charge it with pumice and then hold lip out of way during the polishing, also keeping mouth open till operator uses spray, which will prevent patient getting the powder all over the mouth. Use the round point on the under surface of a bridge, as that surface is generally concave. Where there is no facing (a wash-out bridge) the under surface may be cleaned with a linen strip or wide Cutter's floss charged with pumice. Polish occlusal surfaces with the wheel brush in the engine.

The same technique is used for the lowers, except that the assistant uses the saliva ejector, and endeavors to keep the teeth from being flooded for as long a period as possible or desired.

After all teeth are gone over the disclosing solution should be used to see if any place has been insufficiently polished.

The teeth should now be examined for decay and for faulty contact points, during which the assistant should be polishing partial plates, if the patient has any.

The conversation during the hour should be confined to explaining to patient the proper way of using the floss and to impressing on him the number of surfaces of the tooth there are to be cleaned.

When the operation is completed the proper way of brushing the mouth should be demonstrated by the dentist or the assistant. The only way this can be done is by brushing one's own mouth. The proper make of brush should be prescribed.

As to the fee—it is five dollars an hour of no wasted time. To those who can not afford that fee—about one in three of the people mentioned in the beginning of this paper—it is four dollars an hour, or sixteen dollars a year.

A dentist can afford to do prophylaxis at a smaller fee than he can other dental work, on account of the fact that the prophylactic appointments are made to somewhat suit his convenience, and he can work them in when he is not rushed with other work.

The fee agreed upon to be charged and the length of appointment necessary for each patient should be entered in the book containing the list of prophylactic patients, how often a year they are due, when next wanted, and whether they are to be notified by telephone or letter. This information with any other points the dentist wishes to note. Needless to say this book is not open to inspection by the patient.

The following is a good formula for disclosing solution:

| | |
|---|---|
| Iodin crystals | 50 gr. |
| Potassium iodid | 15 gr. |
| Zinc iodid | 15 gr. |
| Glycerin | 4 dr. |
| Aqua distillata | 4 dr. |

Mix; put up in glass-stoppered bottle.—*Oral Health* for April.

